# Photoperiodic Signaling and Senescence, an Ancient Solution to a Modern Problem?

**DOI:** 10.3389/fpls.2021.634393

**Published:** 2021-03-10

**Authors:** Gloria Serrano-Bueno, Víctor Sánchez de Medina Hernández, Federico Valverde

**Affiliations:** Instituto de Bioquímica Vegetal y Fotosíntesis, CSIC-Universidad de Sevilla, Seville, Spain

**Keywords:** plant development, photoperiod, senescence, flowering, evolution, phytohormones

## Abstract

The length of the day (photoperiod) is a robust seasonal signal originated by earth orbital and translational movements, a resilient external cue to the global climate change, and a predictable hint to initiate or complete different developmental programs. In eukaryotic algae, the gene expression network that controls the cellular response to photoperiod also regulates other basic physiological functions such as starch synthesis or redox homeostasis. Land plants, evolving in a novel and demanding environment, imbued these external signals within the regulatory networks controlling organogenesis and developmental programs. Unlike algae that largely have to deal with cellular physical cues, within the course of evolution land plants had to transfer this external information from the receiving organs to the target tissues, and mobile signals such as hormones were recruited and incorporated in the regulomes. Control of senescence by photoperiod, as suggested in this perspective, would be an accurate way to feed seasonal information into a newly developed function (senescence) using an ancient route (photoperiodic signaling). This way, the plant would assure that two coordinated aspects of development such as flowering and organ senescence were sequentially controlled. As in the case of senescence, there is growing evidence to support the idea that harnessing the reliability of photoperiod regulation over other, more labile signaling pathways could be used as a robust breeding tool to enhance plants against the harmful effects of climate change.

## Introduction

Due to their particular static nature, plants have adapted a high number of interconnected pathways that respond to external and internal stimuli to execute their development programs (Pajoro et al., 2014; Jing and Lin, 2020). Inherently, plants must mature in a plastic way that ensures that their development programs are closely coordinated with the seasonal changes in their environment. In this way, they can optimize all physiological decisions by synchronizing them with the correct time of the year and growth stage (Casal et al., 2004). Each plant species has thus optimized their developmental plans for their particular habitats to maximize growth and the production of offspring. Therefore, to understand and predict plant behavior at any particular physiological stage and organ, we need to interconnect all this information. This could be crucial to protect existing plants or design new varieties capable of coping with the unpredictable weather conditions promoted by global climate change (GCC; Nicotra et al., 2010).

*Arabidopsis thaliana* as an annual model plant has provided a wealth of developmental information, which can be applied to other species, including crops (Ferrier et al., 2011), so this review will focus on annual plants. The advent of the genomic era and the generation of a massive amount of data on plant development from Systems Biology experiments in recent years have increased the need for using computer-aided approaches to handle the accumulated Terabytes of information (Kinoshita and Richter, 2020). However, as already mentioned, this complexity reflects the complex developmental responses of plants to internal and environmental changes. That is why our ability to interconnect different pathways becomes increasingly important to understand the behavior of plants (Franks and Hoffmann, 2012; Majeed et al., 2020; Zhang et al., 2020).

**Figure 1:**
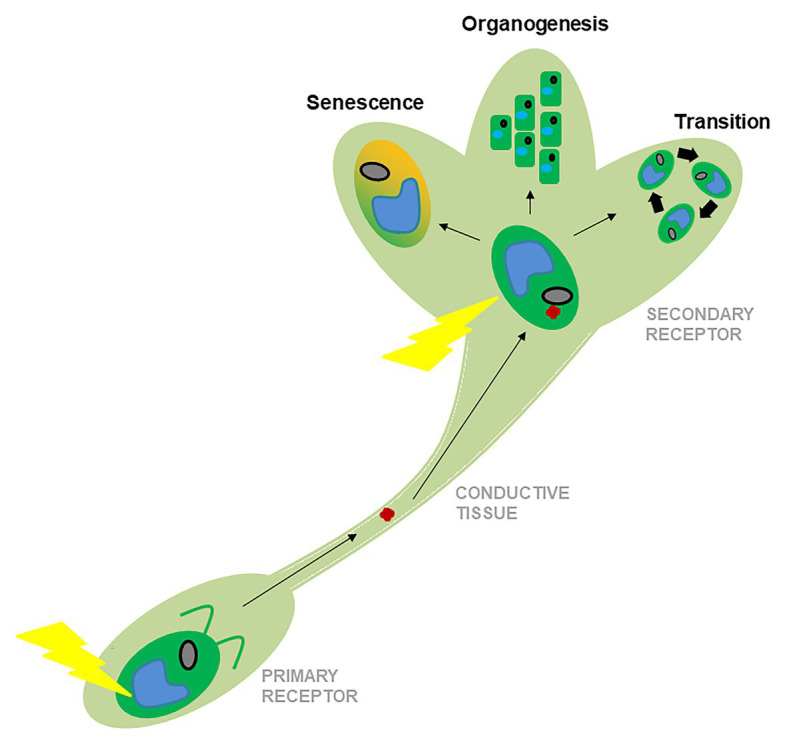
Schematic representation of the evolution of the responses to environmental stimuli from algae to land plants. The primary receptor, like a leave (represented by an algal cartoon, below), responds primarily to physical stimuli (yellow ray). The secondary receptor, like the SAM (represented by a shoot cartoon, above) receives mobile signals that move along the conductive tissues (red dot). After receiving reinforcement from physical stimuli, target organs make different developmental decisions represented here by phase transitions, organogenesis, or senescence.

The correct response to external physical stimuli such as light or temperature is critical for the survival of any organism, and early plants developed a complex gene network to respond successfully to them (Serrano-Bueno et al., 2017; Cheng et al., 2019). With the increasing signaling complexity of the new aerial habitats and the production of new organs (Bowman et al., 2017; de Vries and Archibald, 2018), land plants developed new forms of regulation that included transportable signals such as florigens, tuberigens, signal peptides, and hormones, among other mobile effectors (Thomas et al., 2009; Wang et al., 2015; Briones-Moreno et al., 2017; Figure 1). This may explain why evolutionarily modern and complex developmental programs, such as flower formation or senescence, are deeply intertwined with hormonal signals (Thomas et al., 2009), whereas ancient physiological responses, such as photosynthesis modulation or photoperiodic signaling in the leaf or algae, often respond to more physical stimuli such as changes in light or temperature (Serrano-Bueno et al., 2017). For example, during the flowering process in *Arabidopsis*, there is a relatively low abundance of hormonal regulation in early photoperiodic responses in the leaf, whereas hormones play a more important role in the shoot apical meristem (SAM) and in the stages later in flower development (Lee et al., 2019; Sang et al., 2020).

In this review, we propose a connection between two processes generally considered independently, such as the photoperiodic response and the senescence program (Michelson et al., 2018). Recent results show a seasonal input in the maturation and senescent programs (Körner and Basler, 2010; Kim et al., 2016), which allow us to propose a model through which photoperiod and senescence would be coordinated to ensure a correct developmental program in the plant life cycle.

## Senescence

Senescence is a naturally-occurring phenomenon that involves a gradual decline of functional cells and tissues (Van Deursen, 2014). In many plants, senescence is the final stage in their developmental programs, eventually leading to the death of the organism. However, despite its apparently deteriorating character, it is often a tightly controlled process whose main objective is to allow recycling, remobilization, and reassignment of nutrients from decaying tissues to developing organs (Wen et al., 2020). In plants with short life cycles, this recycling takes place in seeds or fruits, whereas in perennial plants, it mainly happens in storage organs such as stems or roots (Gan and Amasino, 1997; Lim et al., 2007). In annual species, this process provides enough resources for the initiation, progression, and culmination of its reproductive stage, while in perennial species it often implies the beginning of a resting vegetative stage (Woo et al., 2019).

Plant senescence is the result of massive physiological, biochemical, and metabolic changes that take place in all organs, but which have been well described in leaves and flowers. Although essentially dependent on age, senescence occurs when multiple internal and external signals are integrated into age-related information through different regulatory pathways (Buchanan-Wollaston et al., 2003; Lim et al., 2007; Majeed et al., 2020). Considering their given spatial and temporal niches, plants can fine-tune the onset, progression rate, and nature of senescence to ensure successful offspring production and survival. Therefore, senescence is not only a precisely, fine-tuned process for the controlled degradation of macromolecules, but it is also considered a refined evolutionary strategy that plants have acquired to ensure reproduction and survival (Thomas and Stoddart, 1980; Buchanan-Wollaston et al., 2003; Lim et al., 2007; Thomas et al., 2009; Zhang et al., 2020).

### The Senescence Syndrome: Organ-Specific Characteristics

The senescence process involves many morphological, cytological, physiological, and molecular changes that are regulated and carried out following a specific order (Wojciechowska et al., 2018). In this section, we will briefly describe how senescence initiation and progression are regulated at organ-specific level.

#### Leaf Senescence

Leaf senescence is a degenerative process that culminates in the death of leaf cells, and during which they undergo well-defined cell structure and metabolic changes, as well as modifications in gene expression (Lim et al., 2007). Progression of leaf senescence is characterized by a change from assimilation to remobilization of nutrients and the involvement of degenerative events in cellular structures (Masclaux et al., 2000). The earliest cell structural change involves the progressive loss of functionality and breaking down of chloroplasts, where up to 70% of the leaf protein is contained. Concomitantly, a drastic metabolic shift in the chloroplast from anabolism to catabolism takes place, and chlorophyll is massively degraded together with other macromolecules such as RNAs, structural lipids, and proteins. This issue leads to the green-to-yellow color change of leaves that is visible during grain ripening and maturation in crops and during autumn in trees and other perennial plants (Thomas and Stoddart, 1980; Gan and Amasino, 1997; Buchanan-Wollaston et al., 2003; Lim et al., 2007). Unlike chloroplasts, the nucleus and mitochondria remain intact from the onset of senescence until their last stages (Lim et al., 2007). In *Arabidopsis*, mitochondrial integrity and energy production *via* respiration are maintained along the senescence process, although their numbers diminish significantly (Chrobok et al., 2016).

#### Flower Senescence

Flower senescence is the terminal phase of its development and includes flower wilting, blossoms fading, and the shedding of floral sub-structures (Tripathi and Tuteja, 2007). Regulation of flower lifespan is not only essential to ensure that its maintenance is energetically cost-effective for the plant, but also to avoid flowers being misused after fulfilling their role (Ashman and Schoen, 1994; Rogers, 2006). Petals constitute relatively simple organs with similar characteristics to leaves that can be used as a useful model to study the regulation of senescence. The senescence of the petal is the final stage of its development and constitute a tightly regulated programmed cell death process (PCD; Rogers, 2006, 2013; Van Doorn and Woltering, 2008). Although common physiological and biochemical changes are shared between petal and leaf senescence, both processes differ in terms of reversibility, nutrient remobilization purposes, and speed of progression (Ma et al., 2018). Furthermore, flower or petal senescence patterns exhibit a wide variation across species, being flower wilting or withering followed by abscission the most prominent and visibly shown events (Van Doorn and Woltering, 2008; Shahri and Tahir, 2011).

### Hormonal Regulation of Senescence

**Figure 2:**
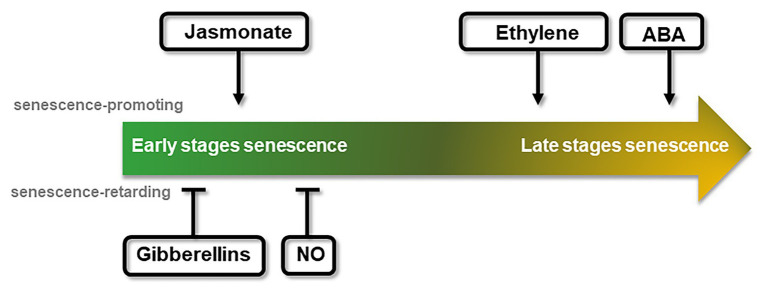
Schematic representation of the chronological effect of different phytohormones on senescence progression. Promotion or delay of senescence can take place at different stages throughout the course of the senescence process, reflected by the central arrow stream. The effect of each hormone or signaling molecule is indicated on the approximate time of their effect. Arrows indicate positive effects over senescence (senescence promoting, above), while bars indicate negative effects (senescence retarding, below).

As plant development progresses, many of the physiological stages are regulated by hormones, often coordinating a complex response. Senescence is not an exception, and many hormones play an important role in the process (Figure 2). With the aim of comparing photoperiod and senescence, this section briefly describes the hormonal control of senescence.

Jasmonate (JA), ethylene, and abscisic acid (ABA) are regarded as senescence-inducing hormones (Jing et al., 2005; Jibran et al., 2013; Hu et al., 2017; Ma et al., 2018). An external addition of methyl jasmonates (MeJA) led to a rapid loss of chlorophyll and photochemical efficiency, as well as to an increased expression of developmental senescence-associated genes (*SAGs*; Xiao et al., 2004; Jung et al., 2007), whereas the expression of photosynthesis-associated genes was reduced (Jibran et al., 2013). JA content was reported to increase during the progress of senescence (He et al., 2002). Consistent with increasing JA levels during leaf and flower senescence, genes involved in JA synthesis and signaling pathways showed an increased expression during organ senescence (Porat et al., 1993, 1995; He et al., 2002; Van Der Graaff et al., 2006; Breeze et al., 2011). Also, a raise in transcript abundance of JA biosynthetic genes has been found previous to any visible signs of chlorophyll loss, suggesting a JA role from early stages of leaf senescence (Figure 2; Jibran et al., 2013). Curiously, the JA-insensitive mutant *coi1-1* from *CORONATINE INSENSITIVE 1* (*COI1*) does not show signs of JA-induced leaf senescence (He et al., 2002), although the repression of *Rubisco activase* (*RCA*) observed in *coi1-1* has been described as a mechanism by which increased JA content can promote senescence (Shan et al., 2011).

In a similar way to JA, ethylene application accelerates leaf and flower senescence, while inhibition of its synthesis or signaling promotes senescence delay (Buchanan-Wollaston et al., 2005; Jing et al., 2005; Kim et al., 2014). Similarly, a reduced expression of the enzyme involved in the ethylene biosynthesis, *1-Aminocyclopropane-1-Carboxylic Acid Oxidase* (*ACO*), delayed flower senescence and flower abscission in some cultivars of petunia, torenia, and carnation (Savin et al., 1995; Aida et al., 1998; Huang et al., 2007; Tan et al., 2014). Mutant plants in ethylene signaling (*ethylene-insensitive2, ein2*) also displayed an arrest in developmental senescence (Buchanan-Wollaston et al., 2005). Another central factor of ethylene signaling, ETHYLENE-INSENSITIVE3 (EIN3), was shown to activate two senescence-promoting transcription factors (TFs), ORE1 and AtNAP, that positively regulate leaf senescence (Kim et al., 2014). Increase of transcript abundance of ethylene synthesis and signaling genes has been found to occur in the same timeframe in which a decline of chlorophyll concentration and transcripts of photosynthetic genes is observed, which suggests that ethylene promotes the latter stages of leaf senescence (Figure 2; Breeze et al., 2011).

As in the case of JA, endogenous ABA levels increase in leaf tissues as they mature, which is accompanied by the upregulation of genes associated with biosynthesis and signaling of ABA (Philosoph-Hadas et al., 1993; Buchanan-Wollaston et al., 2005; He et al., 2005; Van Der Graaff et al., 2006; Breeze et al., 2011). Exogenous application of ABA promotes senescence and abscission (Figure 2; Nooden, 1988; Borochov and Woodson, 1989; Becker and Apel, 1993; Panavas et al., 1998; Yang et al., 2002) and plants under environmental stresses showing leaf senescence have an increased ABA content in their leaves (Lim et al., 2007; Sah et al., 2016). Moreover, ABA regulates the expression of *SAGs* (Zhang and Gan, 2012; Gao et al., 2016; Zhao et al., 2016; Asad et al., 2019). Regarding the phase in which they function, different studies have pointed out to an effect of ABA on leaf senescence that depends on age, concomitant with rising of ABA levels in later stages of flower development. This suggests that ABA may play a role in the enhancement of senescence rather than in its onset (Figure 2; Hunter et al., 2004; Lee et al., 2011; Arrom and Munné-Bosch, 2012; Zhang et al., 2012; Gao et al., 2016).

On the contrary, the phytohormone gibberellin (GA) and the gaseous signaling molecule nitric oxide (NO) have been reported as senescence-retarding effectors whose content declines during the progression of developmental senescence (Figure 2; Schippers et al., 2007; Procházková and Wilhelmová, 2011; Bruand and Meilhoc, 2019). Different studies in GA biosynthesis or GA signaling deficient mutants further support GA role as a negative player in regulating senescence (Van Der Graaff et al., 2006; Chen et al., 2014; Lü et al., 2014). In *Arabidopsis*, expression of the GA deactivating enzyme *GA 2-oxidase 2* was reported to be increased during senescence (Van Der Graaff et al., 2006), while silencing of the GA biosynthetic gene *GA 20-oxidase* resulted in accelerating petal senescence in cut rose (Lü et al., 2014). Leaf senescence in *Arabidopsis* was retarded in the GA biosynthesis mutant *ga1-3*, in which negative regulators of GA signaling pathways abnormally accumulate. Regarding the gaseous signaling NO, exogenous application of NO or NO-donor compounds extended fruits and vegetables post-harvesting life and arrested the senescence of flowers (Leshem et al., 1998). Although NO has been linked to other molecules involved in senescence, no mechanism of NO-preventing effect over leaf senescence has been described yet. Different studies using NO-deficient *Arabidopsis* plants have demonstrated that NO regulates expression of photosynthetic genes and *SAGs* (Mishina et al., 2007; Liu and Guo, 2013). The recent identification of TFs that respond to NO levels in *Arabidopsis* (Imran et al., 2018) can pave the way to further understand how NO contributes to the regulation of senescence.

## Photoperiodic Signaling

In any living organisms, changes in developmental processes throughout the year often define their living strategy. This is particularly true of annual plants, such as *Arabidopsis*, as well many of the crops that feed humanity (rice, corn, and wheat), as they have to precisely plan for germination, growth, reproduction, and senescence to complete a life cycle in 1 year (Preston and Fjellheim, 2020). The way they respond to each seasonal change is also a tactical decision, for example, to coincide with pollinators, outsmart potential opponents, or conversely, modify flowering time to avoid competition (Franks and Hoffmann, 2012). These decisions are closely related to how they respond to fluctuating seasonal changes in environmental conditions and have evolutionarily shaped how their developmental programs respond today.

For a plant, a particularly reliable seasonal change is day length, since its constant change throughout the year establishes the succession of seasons and indicates the duration and intensity of energy availability. Therefore, day length has been used since early in plant evolution as a reliable source of information for making crucial developmental decisions (Serrano-Bueno et al., 2017). In the green alga *Chlamydomonas*, day length (photoperiodic) decisions regulate starch accumulation, reproductive behavior, cell division program (Serrano et al., 2009), photosynthesis protection (Tokutsu et al., 2019), or the retrograde signal from the chloroplast to the nucleus (Gabilly et al., 2019). This evolutionarily conserved mechanism also regulates flowering time and starch synthesis in higher plants, but involves a much larger number of genes, reflecting how evolution often responds to increasingly demanding complexity by amplifying the gene network associated with it (Ortiz-Marchena et al., 2014). But it also indicates that a seasonal detection system based on photoperiod signals was established very early in evolution and still governs many physiological responses in plants (Romero-Campero et al., 2013).

### Early Floral Transition, a Physical Leaf-Triggered Response

**Figure 3:**
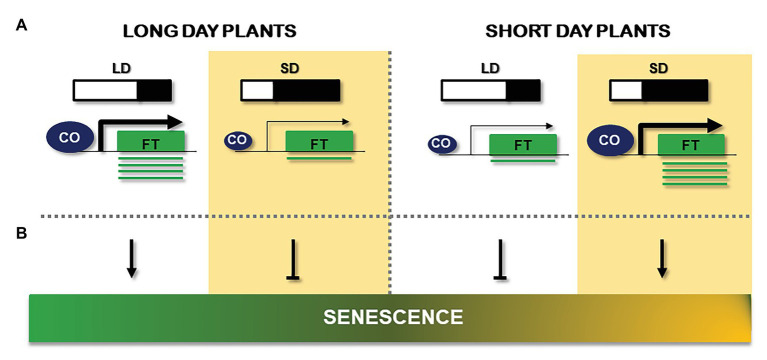
Comparison of the effect of long days (LD) and short days (SD) signals on flowering and senescence. **(A)** Schematic description of day length effect on flowering and its capcity to activate *FT* gene (green box) expression in LD plants (left) and SD plants (right). CONSTANS (CO) abundance is represented by the size of the blue circle, while the arrow size reflects its capacity to activate *FT* mRNA (green line) production. **(B)** Observed results of LD or SD on senescence. Length of the day is represented by a light/black diagram and a white background (LD) or yellow background (SD). Arrows indicate positive effects; bars indicate repressive effects.

One of the best studied photoperiodic responses in higher plants is the floral transition (Andrés and Coupland, 2012; Kinoshita and Richter, 2020). It is becoming increasingly clear that a conserved central module in the developmental processes of plants is designed to receive, process, and transfer signals coming from changes in day length in the leaves to decide the precise moment of the floral transition (Song et al., 2018). This central floral module dates back to gymnosperms, evolved from an ancestral algal system to regulate photoperiodic signaling (Serrano-Bueno et al., 2017), and is conserved in monocots and dicots (Shrestha et al., 2014). The core gene module consists of genes that encode a family of B-Box proteins called BBX or more specifically CONSTANS-like (COL) that can transfer light and time information (from the circadian clock) to the developmental regulatory program (Valverde, 2011; Shim et al., 2017). The presence of these central TFs of which CONSTANS (CO or BBX1) in *Arabidopsis* was the first to be identified (Putterill et al., 1995), must be strictly controlled to assure a perfectly synchronized floral transition (Suárez-López et al., 2001; Valverde et al., 2004). In this way, *CO* expression is controlled at the transcriptional level by a set of TEMPRANILLO (TEM), BHLH (FBHs), and DOF (CDFs) TFs (Castillejo and Pelaz, 2008; Fornara et al., 2009; Ito et al., 2012) whose expression is simultaneously controlled by microRNAs, photoreceptors (PHYs and CRYs), clock genes (GIGANTEA, GI), and LOV-containing ubiquitin ligases (Mizoguchi et al., 2005; Sawa et al., 2007; Kubota et al., 2017), a bryophyte design that is capable of detecting light and sending proteins for degradation through the proteasome (members of the ADAGIO family of E3 ligases such as ZLP or FKF; Song et al., 2014). These set of proteins ensures that *CO* expression is high in the leaves during the day in *Arabidopsis* only in long days (LD) but not in short days (SD; Suárez-López et al., 2001; Figure 3A).

But a simultaneous posttranslational regulatory level is needed to fully confer the day length information to the core photoperiodic floral regulome of the leaf (Valverde et al., 2004; Shim et al., 2017). In this way, CO is controlled at the protein level by a specific association with a set of ring-finger E3 ligases (SPA1, COP1, and HOS1) that are activated through the interaction of CRYs and PHYs, thus transmitting a second light information level to the photoperiod pathway (Jang et al., 2008; Liu et al., 2008; Lazaro et al., 2012). In *Arabidopsis* leaves, stable and active CO protein in the evening of a LD is able to associate with NF-YB, NF-YC TFs, substituting NF-YA from the trimeric conformation (Wenkel et al., 2006). The CO/NF-YB/NF-YC trimeric complex is capable of interacting with DNA and specifying transcriptional activation at CO responsive element (CORE) sites of target promoters, such as the florigen *FT* (Tiwari et al., 2010; Shen et al., 2020) or the starch synthase *GBSSI* (Ortiz-Marchena et al., 2014). In fact, this trimeric conformation is observed in *Chlamydomonas* (Tokutsu et al., 2019) and possibly in other proteins of the CONSTANS, CONTANS-LIKE, TOC1 (CCT) family (Shen et al., 2020). This so-called external coincidence model of CO protein explains why *Arabidopsis* and other long day plants will flower earlier in LD than in SD (Andrés and Coupland, 2012; Yang et al., 2014). But in other short day plants, CO protein has an almost opposite role, functioning as a repressor of *FT* expression in LD and activating *FT* expression in SD as in rice or *Pharbitys* species (Hayama et al., 2003, 2007).

In long day plants, therefore, CO will activate *FT* expression in the leaves during LD and will function as a repressor in SD (Samach et al., 2000; Luccioni et al., 2019), while in short day plants, CO will function in a repressing complex in LD and activate transcription in SD (Hayama et al., 2003; Figure 3A). How CO is able to differentiate both stages and function as a repressor or activator is not fully understood, but it could be at the core of making a plant long day or short day flowering. The so-called neutral plants, which are not able to respond to changes in day length, such as tomato, often present a defective photoperiod response or have lost some of the regulatory components that would respond to light signals (Cao et al., 2015; Gaudinier and Blackman, 2019).

### The Florigen Signal, From Leaves to the SAM

The activation of CO in the leaves is a physical phenomenon that depends upon light density, quality, and exposure length, a complex regulatory mechanism originated from a relatively simple algal toolkit (Serrano-Bueno et al., 2017). When life on land evolved into aerial structures that allowed reproduction independently from water, floral structures, and seeds were created (Pires and Dolan, 2012; Morris et al., 2018), but then, the external information had to be transported from the photosynthetic tissues where it was originated, to the meristems where the reproductive structures were produced. Therefore, different long-distance effectors were designed to transfer developmental and physiological signals from receiving organs to target tissues such as the tuberigen StSP6A (Navarro et al., 2011), the metabolic signal HY5 (Chen et al., 2016), or the clock signal ELF4 (Chen et al., 2020a). In the case of the floral transition, the main florigenic signal is the production of the protein FT in the leaves and its controlled transport to the SAM (Corbesier et al., 2007; Liu et al., 2013). Briefly, the transformation of the vegetative apical meristem into a floral meristem starts with the import of FT into the apical cells *via* the phloem (Abe et al., 2019). Once in the first layers of the SAM, FT can interact with a 14-3-3 chaperon that allows the binding of the TF FLOWERING LOCUS D (FD), and this so-called florigen complex (FC) is then able to activate the expression of other TFs like *SUPRESSOR OF OVEREXPRESSION OF CONSTANS* (*SOC1*), *LEAFY* (*LFY*), or *APETALA1* (*AP1*) that eventually activate the cascade of MADS box TFs producing the different floral whorls (Abe et al., 2005; Wigge et al., 2005; Collani et al., 2019). In fact, QTL analyses have shown that senescence is influenced by functional alleles of the FT repressor, MADS box TF *FLOWERING LOCUS C* (*FLC*), and its positive regulator *FRIGIDA* (*FRI*), whose expression levels negatively correlated with those from senescence-induced genes as well as the floral promoters *FT* and *SOC1* (Wingler, 2011). These studies provide a link between flowering and senescence in *Arabidopsis* independent of photoperiodic signaling.

However, several experiments indicate that CO regulation and function in development maybe more complex than above described, such as participating in an interplay between CO and GA signaling (Andrés et al., 2014), having an active role on stomata opening (Ando et al., 2013) and promoting a link with the circadian clock by the interaction with PRR proteins (Hayama et al., 2017), among others (Kinoshita and Richter, 2020). Recently, a protective role for chloroplast photo redox defense and retrograde signaling has been reported in algae (Gabilly et al., 2019) that could also be conserved in plants, as well as an active role in sugar mobilization from starch (Ortiz-Marchena et al., 2014) and a regulation by phosphorylation (Sarid-Krebs et al., 2015; Chen et al., 2020b). The complex aspects of CO regulation, the different roles it is playing and its presence in different organs suggest that seasonal information is not only controlling floral transition but also other important physiological processes (Valverde, 2011). Here, we present some evidences that suggest that CO may also be involved in senescence by providing a seasonal input to this important developmental process.

## Photoperiod and Senescence

Many physiological processes in plants are affected by photoperiodic signaling, and particularly important for this perspective review, they include flowering and senescence (Nooden et al., 1996; Valverde, 2011). In general terms, *Arabidopsis* developmental processes are accelerated under LD. In this sense, Kim et al. (2016) compared the expression of the senescence marker *SENESCENCE 4* (*SEN4*) in leaves of *Arabidopsis* plants grown under LD and SD conditions. *SEN4* expression increased under both conditions; however, the increase was higher under LD than under SD, suggesting a possible senescence dependence on photoperiod. This effect was also observed in the long day plant Pea (*Pisum sativum* L.). A pea early flowering genetic line named G2, showed early apical senescence under LD, while in SD, it extended the reproductive phase and showed delayed apical senescence (Proebsting et al., 1976, 1978). Parrott et al. (2012) showed an acceleration of leaf senescence associated to LDs in barley (*Hordeum vulgare* L.). Under LD, they observed the beginning of leaf senescence at day 77 after sawing, while under SD treatment, the first symptoms of leaf senescence showed up at day 105. Another point of connection between photoperiod and senescence is mediated by the FBHs TFs. In *Arabidopsis*, overexpression of *FBH4* promoted a high increase in *CO* levels and led to an early flowering phenotype, while *CO* expression was reduced in the *fbh1-4* mutant (Ito et al., 2012). In Petunia flowers (*Petunia hybrida*), *PhFBH4* levels were significantly increased during senescence, indicating a possible connection with photoperiodic signaling (Yin et al., 2015). *PhFBH4* overexpression line showed early flower senescence, whereas *phfbh4* antisense silencing lines extended flower longevity. In addition, the expression of senescence associated genes (*SAG12* and *SAG29*) was drastically altered in Petunia *PhFBH4ox* flowers.

On the contrary, in short day plants, the effect of day length over senescence is the opposite to what was observed in LD. This way, post flowering SD treatment promoted leaf senescence while LD delayed aging in the short day plant Soybean. Han et al. (2006) proposed that the photoperiodic control of development is active from germination through maturation, and the photoperiodic signals are likewise mediated by phytochromes throughout plant development. In rice, the *CO* like gene, *Ghd2* (*Grain number*, *plant height*, and *heading date2*) is involved in the regulation of leaf senescence and drought resistance. The accelerated senescence and the increase of many *SAGs* transcripts in *Ghd2*ox rice plants grown under drought stress revealed the implication of *Ghd2* in drought-induced senescence (Liu et al., 2016).

From the above referred data, it seems that in annual plants, the day length effect over senescence seems to be opposite in long day and short day plants: while LD condition accelerates senescence in long day plants, in short day plants, this process is delayed. On the contrary, SD treatment seems to induce aging in short day plants, whereas reduces senescence in LD plants. Therefore, a correlation between flowering phenotype/CO activity and senescence can be deduced, in both LD and SD plants and this is reflected in Figure 3B. Although an early study in the *Arabidopsis* early flowering accession Ler showed that leaf senescence was unaffected in the *co-2* mutant grown under continuous illumination (Hensel et al., 1993), it has been argued that such light regime could cause the uncoupling of flowering from the senescence process (Wingler, 2011). However, what can be deduced from experiments in the literature run in different light regimes, is that early flowering phenotype and high CO activity are associated with accelerated plant senescence, while late flowering phenotype and low CO activity correlate well with a delay in this effect. These facts reveal that the relationship between photoperiod and senescence may be due in part to CO function.

### Photoperiodic Signaling and Phytohormones

**Figure 4:**
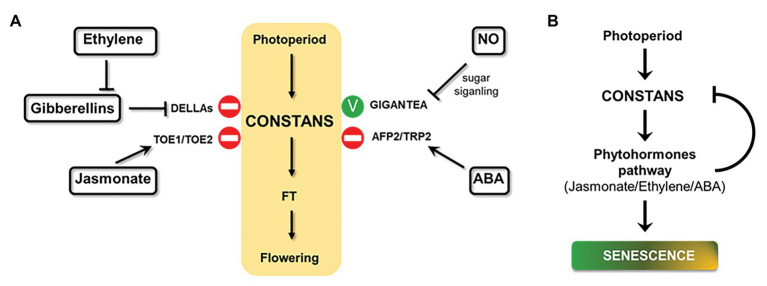
Effect of hormones on photoperiodic signaling and a proposed model for photoperiod and senescence connection. **(A)** Schematic overview of phytohormones effects on central photoperiod module (yellow box) through different effectors (in bold) in *Arabidopsis* plants under LDs (positive effect: green stick and negative effect: stop symbol). **(B)** A model of how photoperiod, phytohormones, and senescence could be related. Arrows indicate positive effects; bars indicate repressive effects.

Phytohormones play an important role in plant senescence as discussed above. This signal also affects flowering time/CO activity in *Arabidopsis* through the photoperiod pathway (Davis, 2009), particularly in late developmental stages (Figure 4). A short description of the effect of phytohormones in photoperiodic flowering follows and will help to understand the relationship between both processes.

It has been described that a mutant of the JA signal receptor CORONATINE INSENSITIVE 1 (*COI1*) showed early flowering phenotype, while overexpression of the JA signal repressor JASMONATE-ZIM-DOMAIN PROTEIN 1 (*JAZ1*) delayed this process (Robson et al., 2010; Zhai et al., 2015). While the loss-of-function *coi1-1* mutant showed early flowering phenotype and high *FT* expression, the molecular mechanism behind the phenotype is unknown (Zhai et al., 2015). Zhang et al. (2015) described that JAZ1 controls the activity of TARGET OF EAT1 (TOE1) and TOE2, which repress *FT* transcription. In the morning, TOE1 and TOE2 can form a complex with CO, while in the afternoon, both proteins interact with CO stabilizer FLAVIN-BINDING, KELCH REPEAT, F-BOX1 (FKF1) to suppress CO activity in both cases (Song et al., 2012; Zhang et al., 2015). Regarding the effect of photoperiod over JA pathway, Cagnola et al. (2018) analyzed the transcriptome of *Arabidopsis* plants grown under SD and then transferred to LD. The study revealed that LD enhanced JA response to increase plant defense; however, this effect was independent of the hormone levels.

Among phytohormones, the role of GAs in *Arabidopsis* flowering is probably the best understood (Davis, 2009). Exogenous application of GAs as well as overexpression of the biosynthetic gene *GA5* promoted flowering (Huang et al., 1998; Coles et al., 1999). Diverse genetic studies also suggested that GA signaling promoted flowering under both SD and LD (Galvão et al., 2012; Porri et al., 2012; Yu et al., 2012). Two studies published in 2016 revealed that GAs induce *FT* expression by a CO-dependent pathway. Moreover, DELLA proteins, the main repressors of GA signaling, can directly interact with CO and inhibit CO/FT-mediated flowering in LDs (Wang et al., 2016; Xu et al., 2016). These facts evidence an integration of GA pathway and photoperiodic signaling to modulate flowering under LDs. Regarding flowering induction by GAs in SD, a recent study stablished that MYC3, a bHLH TF, is stabilized by DELLA proteins in the GA pathway to suppress *FT* expression by opposing CO activation (Bao et al., 2019). MYC3 regulates flowering under SD through *FT* suppression. This TF competes with CO to regulate *FT* transcription. Therefore, GAs promote flowering in SD through DELLA proteins interaction with MYC3. This interaction promotes GA-mediated degradation of MYC3, releasing CO/FT-mediated flowering in SD (Bao et al., 2019).

Ethylene production results in a delayed floral transition. *Arabidopsis* plants grown in the presence of ethylene or a precursor, showed late flowering phenotype (Achard et al., 2006). Similarly, the *Arabidopsis* mutant *ctr1*, a main negative regulator of ethylene signaling, showed the same flowering phenotype under LD and SD photoperiodic conditions (Achard et al., 2007). This delay of flowering can be partially rescued by mutation of genes encoding DELLAs. This finding indicates that the effect of ethylene on flowering may be in part due to modulating the activity of DELLA proteins. Also, activated ethylene signaling enhanced the accumulation of DELLAs by reducing bioactive GA levels (Achard et al., 2007). It has been demonstrated that the effect of ethylene on flowering depends on EIN3, so that ethylene stabilizes EIN3 and EIN3-like proteins by inhibiting the activity of their proteases Cullin1-based E3 complexes EBF1/EBF2 (Binder et al., 2007). Actually, GAs application partially restored flowering time in *ebf1ebf2* double mutant, indicating that ethylene effect over flowering cannot be exclusively due to the inhibition of GA signaling. This fact also reveals the existence of an unknown ethylene control of flowering independent of DELLAs (Achard et al., 2007). Regarding the effect of photoperiod over ethylene pathway, an early target gene of CO activity is involved in ethylene biosynthesis (Samach et al., 2000). *ACS10*, which encodes a putative synthase involved in ethylene biosynthesis, was differentially expressed in the CO-activity inducible plant *35S:CO:GR* in response to the inducer DEXAMETASONE (DEX). Treatment of *35S:CO:GR* plants with DEX increased the abundance of *ACS10* mRNA (Samach et al., 2000).

Abscisic acid signal delays flowering by upregulating *FLC* and downregulating *CO* through ABI5-BINDING PROTEIN 2 (AFP2). The bZIP TF ABSCISIC ACID-INSENSITIVE MUTANT 5 (ABI5) can bind to the *FLC* promoter, activate *FLC* expression and delay flowering (Wang et al., 2013). Furthermore, flowering time was significantly delayed, and *CO* expression was reduced, in an *Arabidopsis AFP2ox* line under LD conditions, while, in the loss-of-function *afp2* mutant, flowering time was markedly accelerated and *CO* expression was increased. This study showed that AFP2 interacts with CO and the transcriptional corepressor TOPLESS-related protein2 (TPR2) to form the CO-AFP2-TPR2 complex that mediates CO degradation during the night (Chang et al., 2019). These studies reveal a role for AFP2 in photoperiodic flowering by modulating CO levels. Regarding the effect of photoperiod over ABA pathway, Zeevaart (1971) analyzed ABA content in the LD plant spinach (*Spinacia oleracea* L.) transferred from SD to LD, and the ABA content increased 2 to 3-fold.

It has been described that the gaseous signaling molecule NO repressed flowering in *Arabidopsis* (He et al., 2004). Plants treated with a NO-promoting agent, as well as a mutant overproducing NO (*nox1*), showed late flowering phenotype, while the *Arabidopsis* NO deficient mutant (*nos1*) flowered early. *nox1* mutant showed upregulation of the *FLC* transcript; however, the molecular mechanism still awaits further investigation. Late flowering phenotype associated to NO levels cannot be exclusively assigned to the interaction with the vernalization pathway, since expression levels of the photoperiodic genes *CO* and *GI* were reduced in the *nox1* mutant (He et al., 2004). Thus, NO was proposed to interact with the photoperiod pathway to regulate *CO* expression through a GI-dependent pathway. A recent study revealed that this interaction depends on sugar signaling (Zhang et al., 2019). Sucrose supplementation reversed the effects of NO treatment over *CO* and *GI* transcripts. While NO induced S-nitrosation modification on CO and GI, sucrose reduced the levels of this modification in both proteins (Figure 4A).

## Concluding Remarks

The influence of phytohormones on the central regulatory module of photoperiodic signaling often involves the central hub CONSTANS, as shown in Figure 4A. Interestingly, many senescence-inducing phytohormones, such as ethylene, jasmonato, and ABA, are also involved in the photoperiodic-dependent flowering signal. In these cases, phytohormones delay flowering time by activating CO-repressors, DELLAs, TOE1/TOE2, and AFP2/TRP2 proteins. Although there are many works that analyze the effects of phytohormones over the photoperiodic pathway, few of them describe the effect of photoperiodic signaling on phytohormones and their responses in *Arabidopsis* (Samach et al., 2000; Cagnola et al., 2018), or in other species (Zeevaart, 1971). It would be of particular interest to further investigate this relationship in order to establish a complete overview of photoperiod, phytohormones, and senescence cycle in plants.

Most of the studies we have referred to in this review have been conducted on the annual model plant *Arabidopsis*. It is not clear whether photoperiodic regulation of senescence could also occur in perennials. It has been reported that branches of the perennial *Arabis alpina* could behave as annuals, since leaves from flower-containing branches senesced earlier than those from flower-devoid branches (Wingler, 2011). Another major question concerns the directionality of the connection between flowering and senescence in perennials, since flower promotion does not always result in early senescence. Miryeganeh et al. (2018) showed that senescence is synchronized in *Arabidopsis* regardless of flowering initiation; however, a strong synchronization of flowering termination and whole-plant senescence was observed. Senescence-related genes were upregulated before flowering termination, pointing out that nutrient remobilization preceded reproduction termination (Miryeganeh et al., 2018). Further studies will be needed to explore whether in perennials flowering and senescence are connected in a similar manner to annuals.

Based on all the data collected in this perspective article, we propose a model on how photoperiod and senescence could be related, where CO regulation could be the central axis (Figure 4B). This model includes the implication of phytohormones, such as JA, ethylene, and ABA, on this relationship. In this model, we suggest that senescence regulation by photoperiod is due to CO activation of phytohormone responses. This scenario also includes a negative feedback loop, where phytohormones, in turn, inhibit CO activity.

In conclusion, this perspective review tries to shed new light on the increasingly complex regulation of plant development by integrating two independent, but chronologically interconnected programs, such as photoperiodic signaling and senescence. Early physiological responses (light and temperature) would be transmitted through physical signaling systems of archaic origin, while more complex regulatory pathways of modern origin would involve mobile signals and/or hormonal actions. Developing genetic strategies to modulate robust and constant photoperiodic signals to control plant development could have the added value of balancing the deleterious effects that other less consistent signals such as temperature, drought, or salinity will have on plant growth in the future GCC scenario.

## Author Contributions

FV and GS-B designed the manuscript and figures. All authors contributed to the article and approved the submitted version.

### Conflict of Interest

The authors declare that the research was conducted in the absence of any commercial or financial relationships that could be construed as a potential conflict of interest.
